# The Contributions of Gamma Probe to Lesion Detectability and Surgical Safety in Recurrent Thyroid Cancer at Risk

**DOI:** 10.4274/Mirt.148

**Published:** 2013-08-01

**Authors:** Salih Sinan Gültekin, Güleser Saylam, Tuncay Delibaşı, Hakan Korkmaz

**Affiliations:** 1 Dışkapı Yıldırım Beyazıt Training and Research Hospital, Division of Nuclear Medicine, Ankara, Turkey; 2 Dışkapı Yıldırım Beyazıt Training and Research Hospital, Department of Otolaryngology-Head and Neck Surgery, Ankara, Turkey; 3 Dışkapı Yıldırım Beyazıt Training and Research Hospital, Department of Endocrinology and Metabolism, Ankara, Turkey

**Keywords:** technetium Tc 99m sestamibi, thyroidectomy, local neoplasm recurrence, thyroid cancer, Scintillation Counting

## Abstract

In patients, who underwent thyroid surgery or treated with I-131 radioiodine previously for differentiated thyroid cancer, a second surgical intervention carries higher risks due to distortion of the natural anatomy and development of fibrotic/cicatricial tissue. In addition, accurate assessment of current status about extent of the disease is important in terms of success of the surgery. In this case report, we present the positive contribution of intraoperative gamma probe used for lesion detectability and for surgical safety in a patient operated for several times and administered high cumulative dose of radioiodine therapy for diffentiated thyroid carcinoma previously.

**Conflict of interest:**None declared.

## INTRODUCTION

Differentiated thyroid cancer (DTC) usually has a good prognosis following an appropriate initial treatment (1). Loco-regional recurrence can occur in 20% of treated DTC patients in the follow-up period (2). The main treatment modality is surgery but a protocol combining radioiodine therapy and surgery is often applied. Loss of 131I-radioiodine avidity can be seen in patients who have been treated with repeated applications of the 131I-radioiodine therapy (RIT). Neck ultrasonography (US), ^99m^Tc-methoxyisobutylisonitrile (^99m^Tc-MIBI) and ^18^F-fluorodeoxyglocose (^18^F-FDG) scans and intraoperative gamma probe (GP) are shown to be useful in such patients in varying proportions (3,4,5,6,7). The accurate identification of recurrent tumor tissues may be compromised after repeated operations. Surgical GP technique has some potential advantages and it may offer some benefits in the previously operated neck regions (8,9,10). In a comparative way with other imaging methods, we present several contributions of intraoperative gamma probe method for lesion detectability and about surgical safety in a patient with recurrent thyroid carcinoma whose reoperation is risky because of previous applications.

## CASE REPORT

**Figure 1:**
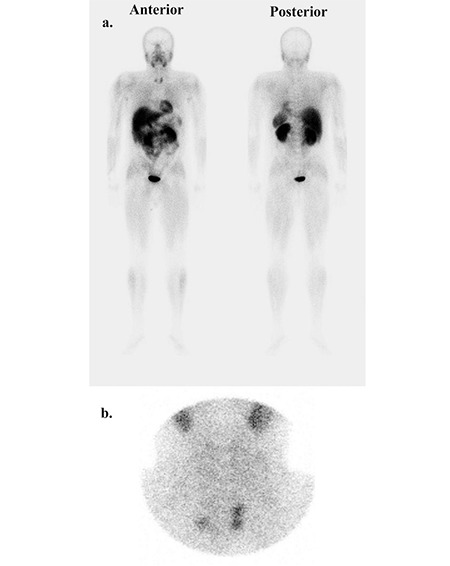
Preoperative 99mTc-MIBI whole body scan (a) and neck pinholeimage (b) show pathological uptakes in a focus in the right thyroid bedand two foci in the left thyroid bed

**Figure 2:**
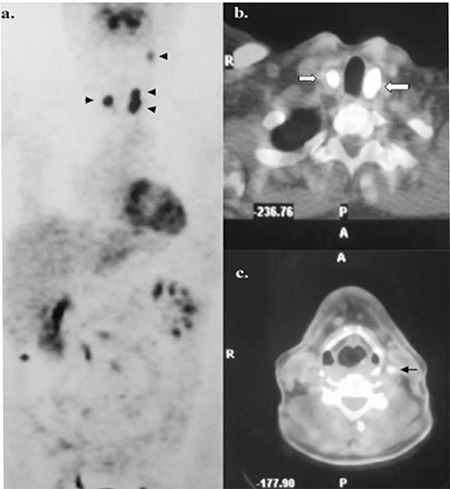
Preoperative 18F-FDG whole-body PET (a) and axial PET-CTfusion (b, c) images show pathological uptakes (arrowsheads) in boththyroid beds (white arrows) and a left jugular lymph node (black arrow).

A 54-year-old male patient with DTC, had a history of multiple of thyroid and neck operations with repetitive RIT (total 37 GBq) during the 15-year follow-up period in other centers. After the last RIT, whole-body scan was normal but he underwent left limited neck dissection for suspicious fine-needle cytology and histopathological examination revealed papillary carcinoma metastases. Then he applied to our center; he was under L-thyroxine suppression therapy, serum thyroglobulin, antithyroglobulin-antibody and thyroid stimulating hormone (TSH) levels were, 21.53 ng/mL, 57 U/mL and <0.003 uIU/mL, respectively. The neck US revealed lesions with dimensions of 14x14x17 mm in right thyroid bed, 10x10x15 mm in left thyroid bed and a lymph node with dimensions of 6x10x10 mm in left cervical region. A 99mTc-MIBI scan revealed three pathological foci in the thyroid bed (Figure 1), 18F-FDG positron emission tomography-computed tomography (PET-CT) scan revealed increased uptakes in right thyroid bed (_SUV_max: 12.5), left thyroid bed (_SUV_max: 14.8) and in a left jugular lymph node (_SUV_max: 6.0) (Figure 2). 

**Figure 3:**
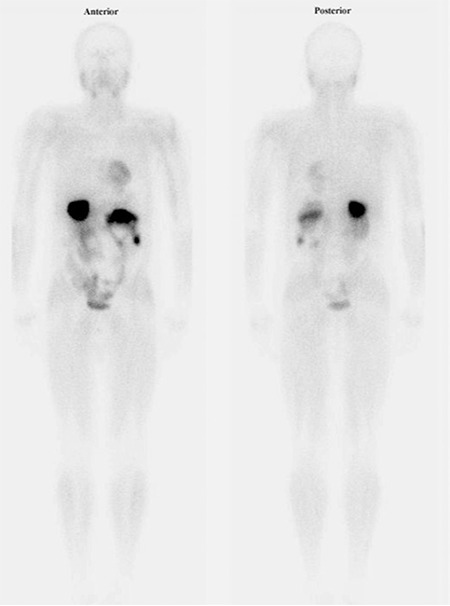
Postoperative 99mTc-MIBI whole body scan shows that thereis not any pathological uptake and physiological radiotracer distributionis observed.

The decision for treatment was re-operation of the patient with the surgical gamma probe guidance. Ten minutes before incision, 37 MBq of 99mTc-MIBI was injected intravenously. The exploration was difficult due to dense fibrosis of the surgical field and anatomical distortions. Bilaterally recurrent laryngeal nerves were identified and preserved, but parathyroid glands were not encountered. Radioactivity counts were collected intraoperatively with a surgical GP (Europrobe III, Eurorad, France) for background (BG), and hot spots which indicated the pathological lesion (L). GP counts revealed five hot spots; on the right thyroid bed (L/BG: 3.3), on the left thyroid bed (L/BG: 4.4 and 3.8), on the left cervical level 2 (L/BG: 3.6) and level 3 (L/BG: 3.2). There was a statistically significant difference between mean L/BG values before excision (3.66±0.48) and after excision (1.40±0.27). All condemned tissues were removed completely, and the postoperative period was uneventful. The patient was discharged next day with mobile cords and normal calcium level. Histopathological examination revealed papillary carcinoma foci in both thyroid beds (>10 mm), two conglomerate metastatic lymph nodes in left thyroid bed (>10 mm), and two metastatic lymph nodes at the level 2 and 3 (<10 mm). When the patient was controlled on the eight months after the surgery, 99mTc-MIBI scan (Figure 3) and chest CT findings were normal, serum Tg and TSH levels were 6.30 ng/ml and 0.02 uIU/mL, respectively.

**Literature Review and Discussion**

Local-regional recurrences may develop in up to 20% of patients with DTC. They are also associated with a reduction in survival and an increase in mortality in some series (2,3). Although recurrence usually occurs during the early follow-up period, it can also rarely occur later in the follow-up as seen in our case. When the lymph node recurrences are located in central compartment, or behind the vascular structures, or small in size; they may not be palpable and usually diagnosed with the combination of elevated serum Tg levels and neck US (3,11).

Loss of ^131^I-radioiodine avidity by metastatic thyroid cells may develop as a result of progressive tumor dedifferentiation (6). This can be partly explained by a decrease in sodium iodide symporter expression after RITs. Although conventional ^131^I-radioiodine applications become useless, serum Tg levels can still be a useful marker in such DTC patients, because it reflects different cellular mechanisms (6,7). When loco-regional recurrence is detected in patients with elevated Tg levels and negative ^131^I-radioiodine scan, accurate diagnosis of localization and spread of the disease is crucial and US, ^99m^Tc-MIBI and ^18^F-FDG PET-CT scans preoperatively and radioguided surgery intraoperatively might be helpful for this purpose (4,5,6,7,12). Casara et al. (4) found a good sensitivity for 99mTc-MIBI scan (94,1%) and neck US (90,2%) in the determination of cervical lymph node metastases from DTC. On the other hand, Rubello et al (7) showed that only 58% of the foci identified by a pre-operative 99mTc-MIBI scan were metastatic. Rubello et al. (6) emphasized that all lesions seen in high-resolution US were also identified with GP-guided surgery. Nahas et al (12) obtained sensitivity of only 66% but with a high specificity and positive predictive value (100%) when they evaluated the impact of 18F-FDG PET-CT in 33 patients with suspected recurrent papillary thyroid cancer. In another study by Razfar et al. (5), they found a sensitivity of 80.7%, specificity of 88.9%, positive predictive value of 94.7% for 18F-FDG PET-CT scan in the setting of recurrent disease from DTC in 125 patients. The surgery is the treatment of choice but re-operation has its own inherent technical difficulties because of previous surgical interventions (8). Success rate of re-operation may decline due to the distortion of natural anatomical structures, development of fibrotic/scatrical tissues and decreased volume of residual or metastatic tissues. Thus, intraoperative quick labeling and correct identification of functional tissues with an appropriate radiotracer can be helpful. Radioguided surgery was assessed very useful in 14/58 patients, useful in 22/58 patients, moderately useful in 17/58 patients and not useful in 5/58 patients in the study of Rubello et al (7). 

We used intraoperative GP technique with low dose 99mTc-MIBI (13) in a patient with history of multiple thyroid surgeries and high dose RIT. ^99m^Tc-MIBI is a radiopharmaceutical with convenient specifications such as; easy access, low cost and high counting statistics. Utilization of low dose protocol provides considerably reduced radiation dose received by the fingers of the surgeon. Use of the same radiopharmaceutical in the preoperative and intraoperative assessment supports reliability and confirmation of the findings. Serum Tg levels usually decrease after removal of tumoral or remnant tissues. A 1 or 2 ng/ml Tg cutoff value can be used for the follow-up of disease-free status in the post-operative period. Rubello et al (7) found that there were normal serum Tg levels (<2 ng/ml) in 43/58 patients, slightly high Tg levels (<10 ng/ml) in 12/58 patients and extremely high Tg levels in 3/58 patients, within the mean follow-up of approximately 30 months. In our patient in the eighth month after surgery, serum Tg level was 6.30 ng/ml and there was no evidence of metastatic disease on the imaging studies. This condition was considered in accordance with presence of microscopic disease. 

In conclusion, with surgical GP technique, recurrent/residual neoplastic thyroid tissues had been removed with high precision without any complications in a technically difficult surgical field. Additionally, on the basis of histopathological evaluation we observed that GP method with low dose 99mTc-MIBI contributed to preoperative studies for lesion detectability by determining an additional occult lymph node metastases.
